# (*E*)-5,5′-(Diazene-1,2-di­yl)diisophthalic acid *N*,*N*-dimethyl­formamide disolvate

**DOI:** 10.1107/S1600536808032819

**Published:** 2008-10-25

**Authors:** Li Zhang, Zhi-Rong Qu

**Affiliations:** a Ordered Matter Science Research Center, College of Chemistry and Chemical Engineering, Southeast University, Nanjing 210096, People’s Republic of China

## Abstract

The title compound, C_16_H_10_N_2_O_8_·2C_3_H_7_NO, was synthesized by the reductive condensation reaction of 5-nitro­isophthalic acid in the presence of NaOH. The tetra-acid mol­ecule, which has a crystallographically imposed centre of symmetry, adopts an *E* configuration with respect to the azo group. In the crystal packing, mol­ecules are linked through inter­molecular O—H⋯O and C—H⋯O hydrogen-bonding inter­actions, forming chains propagating in [2
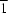
0].

## Related literature

For general background information on the applications of azo compounds, see: Chung & Stevens (1993 ▶); Carliell *et al.* (1995 ▶).
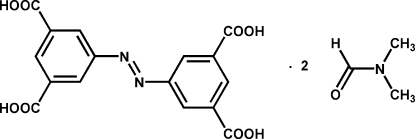


## Experimental

### 

#### Crystal data

C_16_H_10_N_2_O_8_·2C_3_H_7_NO
*M* *_r_* = 504.45
Triclinic, 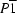

*a* = 6.2926 (13) Å
*b* = 7.2114 (13) Å
*c* = 13.653 (4) Å
α = 80.94 (4)°
β = 85.30 (4)°
γ = 81.72 (3)°
*V* = 604.3 (3) Å^3^
*Z* = 1
Mo *K*α radiation
μ = 0.11 mm^−1^
*T* = 293 (2) K
0.20 × 0.20 × 0.20 mm

#### Data collection

Rigaku SCXmini diffractometer
Absorption correction: multi-scan (*CrystalClear*; Rigaku, 2005 ▶) *T*_min_ = 0.971, *T*_max_ = 0.979
5593 measured reflections
2363 independent reflections
1607 reflections with *I* > 2σ(*I*)
*R*_int_ = 0.029

#### Refinement

*R*[*F* ^2^ > 2σ(*F* ^2^)] = 0.052
*wR*(*F* ^2^) = 0.154
*S* = 1.04
2363 reflections
167 parameters
H-atom parameters constrained
Δρ_max_ = 0.20 e Å^−3^
Δρ_min_ = −0.19 e Å^−3^

### 

Data collection: *CrystalClear* (Rigaku, 2005 ▶); cell refinement: *CrystalClear*; data reduction: *CrystalClear*; program(s) used to solve structure: *SHELXS97* (Sheldrick, 2008 ▶); program(s) used to refine structure: *SHELXL97* (Sheldrick, 2008 ▶); molecular graphics: *SHELXTL* (Sheldrick, 2008 ▶); software used to prepare material for publication: *SHELXL97* and *PRPKAPPA* (Ferguson, 1999 ▶).

## Supplementary Material

Crystal structure: contains datablocks I, global. DOI: 10.1107/S1600536808032819/rz2248sup1.cif

Structure factors: contains datablocks I. DOI: 10.1107/S1600536808032819/rz2248Isup2.hkl

Additional supplementary materials:  crystallographic information; 3D view; checkCIF report

## Figures and Tables

**Table 1:** Hydrogen-bond geometry (Å, °)

*D*—H⋯*A*	*D*—H	H⋯*A*	*D*⋯*A*	*D*—H⋯*A*
O1—H1⋯O5^i^	0.82	1.72	2.541 (2)	174
O3—H3⋯O2^ii^	0.82	1.94	2.697 (2)	154
C4—H4⋯O3^ii^	0.93	2.42	3.305 (2)	159
C11—H11⋯O2^iii^	0.93	2.58	3.240 (3)	128

Symmetry codes: (i) 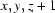; (ii) 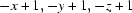; (iii) 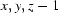.

**Fig. 1.**
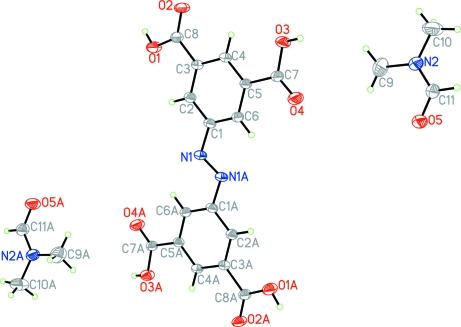
The molecular structure of the title compound with the atomic numbering scheme. Displacement ellipsoids were drawn at the 30% probability level. [Symmetry code: (A) -x+3, -y, -z+1].

**Fig. 2.**
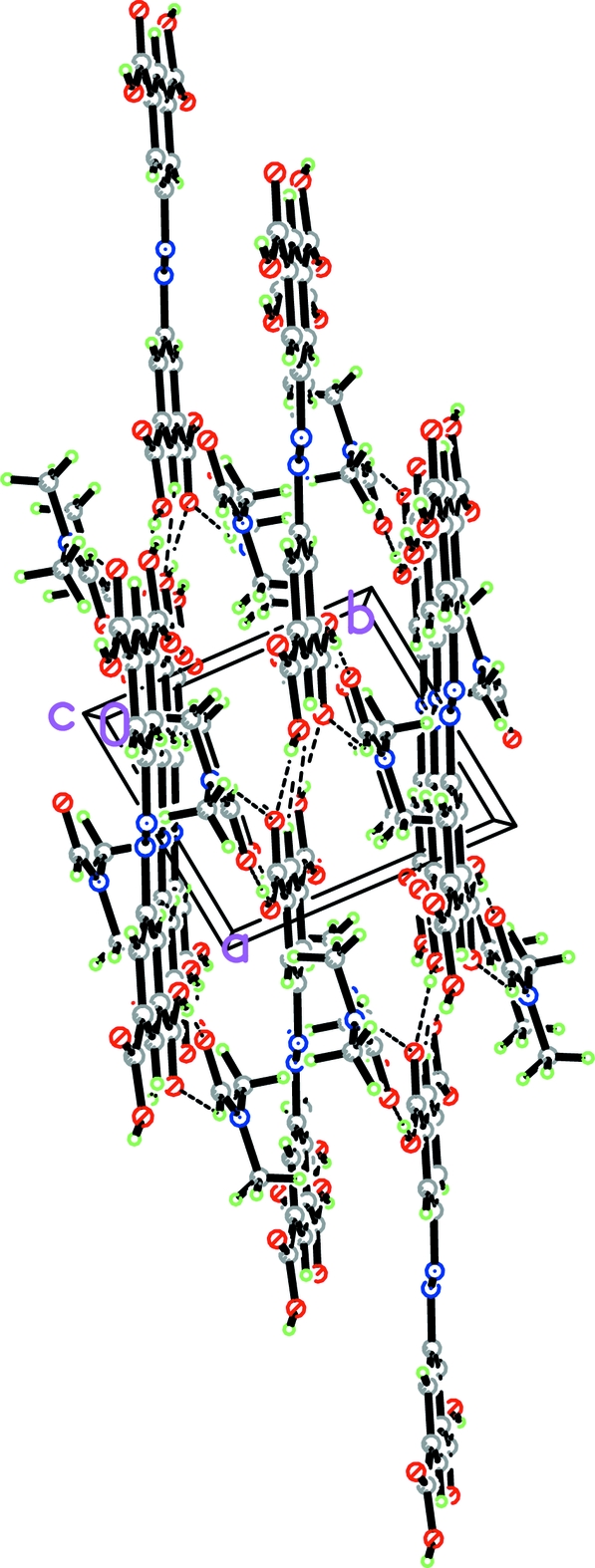
Packing diagram of the title compound, showing the structure along the c axis. Intermolecular hydrogen bonds are shown as dashed lines.

## supplementary crystallographic information

### Comment

Azo compounds are used as dyes in textile, paper manufacturing, pharmaceutial and food industries (Chung & Stevens, 1993; Carliell *et al.*, 1995). Herein, we report the crystal structure of the title compound, which was obtained by reductive condensation reaction of 5-nitroisophthalic acid in the presence of NaOH.

The acid molecule of the title compound (Fig. 1) has a crystallographically imposed centre of symmetry and adopts an E-configuration with respect to the azo group. The molecular conformation is stabilized by intramolecular C—H···O hydrogen bonds (Table 1). In the crystal packing (Fig. 2), molecules are linked into layers parallel to the (210) plane by intermolecular O—H···O and C—H···O hydrogen bonds (Table 1).

### Experimental

A solution of sodium hydroxide (35.9 g, 0.9 mol) in H_2_O (125 ml) was added dropwise to a suspension of 5-nitroisophthalic acid (10 g, 50.3 mmol) in H_2_O (125 ml). The mixture was heated at 50°C for 18 h. After filtration, the yellow solid obtained was dissolved in H_2_O and acidified with HCl. Crystals suitable for X-ray analysis were obtained after 10 days by slow evaporation of a DMF solution.

### Refinement

All H atoms were positioned geometrically and were allowed to ride on their parent atoms,  with C—H = 0.93-0.96 Å, O—H = 0.82 Å, and with *U*_iso_(H) = 1.5*U*_eq_(C, O) or 1.2*U*_eq_(C) for aromatic and aldehyde H atoms.

### Crystal data

**Table:**

C_16_H_10_N_2_O_8_·2C_3_H_7_NO	*Z* = 1
*M**_r_* = 504.45	*F*(000) = 264
Triclinic, *P*1	*D*_x_ = 1.386 Mg m^−^^3^
Hall symbol: -P 1	Mo *K*α radiation, λ = 0.71073 Å
*a* = 6.2926 (13) Å	Cell parameters from 1381 reflections
*b* = 7.2114 (13) Å	θ = 2.9–27.4°
*c* = 13.653 (4) Å	µ = 0.11 mm^−^^1^
α = 80.94 (4)°	*T* = 293 K
β = 85.30 (4)°	Cuboid, colourless
γ = 81.72 (3)°	0.20 × 0.20 × 0.20 mm
*V* = 604.3 (3) Å^3^	

### Data collection

**Table:**

Rigaku SCXmini diffractometer	2363 independent reflections
Radiation source: fine-focus sealed tube	1607 reflections with *I* > 2σ(*I*)
graphite	*R*_int_ = 0.029
Detector resolution: 13.6612 pixels mm^-1^	θ_max_ = 26.0°, θ_min_ = 2.9°
ω scans	*h* = −7→7
Absorption correction: multi-scan (CrystalClear; Rigaku, 2005)	*k* = −8→8
*T*_min_ = 0.971, *T*_max_ = 0.979	*l* = −16→16
5593 measured reflections	

### Refinement

**Table:**

Refinement on *F*^2^	Primary atom site location: structure-invariant direct methods
Least-squares matrix: full	Secondary atom site location: difference Fourier map
*R*[*F*^2^ > 2σ(*F*^2^)] = 0.052	Hydrogen site location: inferred from neighbouring sites
*wR*(*F*^2^) = 0.154	H-atom parameters constrained
*S* = 1.04	*w* = 1/[σ^2^(*F*_o_^2^) + (0.0869*P*)^2^ + 0.0094*P*] where *P* = (*F*_o_^2^ + 2*F*_c_^2^)/3
2363 reflections	(Δ/σ)_max_ < 0.001
167 parameters	Δρ_max_ = 0.20 e Å^−^^3^
0 restraints	Δρ_min_ = −0.19 e Å^−^^3^

### Special details

**Table:**

Geometry. All e.s.d.'s (except the e.s.d. in the dihedral angle between two l.s. planes) are estimated using the full covariance matrix. The cell e.s.d.'s are taken into account individually in the estimation of e.s.d.'s in distances, angles and torsion angles; correlations between e.s.d.'s in cell parameters are only used when they are defined by crystal symmetry. An approximate (isotropic) treatment of cell e.s.d.'s is used for estimating e.s.d.'s involving l.s. planes.
Refinement. Refinement of *F*^2^ against ALL reflections. The weighted *R*-factor *wR* and goodness of fit *S* are based on *F*^2^, conventional *R*-factors *R* are based on *F*, with *F* set to zero for negative *F*^2^. The threshold expression of *F*^2^ > σ(*F*^2^) is used only for calculating *R*-factors(gt) *etc*. and is not relevant to the choice of reflections for refinement. *R*-factors based on *F*^2^ are statistically about twice as large as those based on *F*, and *R*- factors based on ALL data will be even larger.

### Fractional atomic coordinates and isotropic or equivalent isotropic displacement parameters (Å^2^)

**Table:**

	*x*	*y*	*z*	*U*_iso_*/*U*_eq_	
C1	1.2504 (3)	0.1247 (3)	0.53879 (14)	0.0368 (5)	
C2	1.1485 (3)	0.1486 (3)	0.63147 (14)	0.0388 (5)	
H2	1.2213	0.1037	0.6889	0.047*	
C3	0.9387 (3)	0.2393 (3)	0.63829 (14)	0.0362 (4)	
C4	0.8321 (3)	0.3072 (3)	0.55177 (14)	0.0361 (5)	
H4	0.6909	0.3664	0.5558	0.043*	
C5	0.9357 (3)	0.2870 (2)	0.45901 (13)	0.0340 (4)	
C6	1.1450 (3)	0.1962 (2)	0.45217 (14)	0.0360 (4)	
H6	1.2144	0.1830	0.3903	0.043*	
C7	0.8248 (3)	0.3644 (3)	0.36581 (14)	0.0393 (5)	
C8	0.8253 (3)	0.2634 (3)	0.73691 (14)	0.0429 (5)	
C9	0.5021 (5)	0.1873 (5)	0.1616 (2)	0.0951 (10)	
H9A	0.6554	0.1852	0.1536	0.143*	
H9B	0.4689	0.0633	0.1894	0.143*	
H9C	0.4427	0.2763	0.2052	0.143*	
C10	0.1791 (4)	0.2888 (5)	0.0659 (2)	0.0897 (10)	
H10A	0.1367	0.3194	−0.0013	0.135*	
H10B	0.1337	0.3953	0.1003	0.135*	
H10C	0.1133	0.1817	0.0988	0.135*	
C11	0.5328 (4)	0.2465 (4)	−0.01670 (17)	0.0583 (6)	
H11	0.4662	0.2823	−0.0764	0.070*	
N1	1.4637 (2)	0.0230 (2)	0.54104 (12)	0.0396 (4)	
N2	0.4107 (3)	0.2433 (3)	0.06578 (13)	0.0560 (5)	
O1	0.9368 (2)	0.1852 (3)	0.81263 (10)	0.0609 (5)	
H1	0.8628	0.1957	0.8643	0.091*	
O2	0.6425 (2)	0.3456 (2)	0.74637 (11)	0.0587 (5)	
O3	0.6225 (2)	0.4372 (2)	0.38429 (11)	0.0559 (5)	
H3	0.5702	0.4880	0.3321	0.084*	
O4	0.9084 (2)	0.3631 (3)	0.28388 (11)	0.0658 (5)	
O5	0.7304 (3)	0.2054 (3)	−0.02061 (11)	0.0725 (6)	

### Atomic displacement parameters (Å^2^)

**Table:**

	*U*^11^	*U*^22^	*U*^33^	*U*^12^	*U*^13^	*U*^23^
C1	0.0248 (10)	0.0368 (10)	0.0480 (12)	−0.0002 (7)	0.0004 (8)	−0.0085 (9)
C2	0.0308 (10)	0.0441 (11)	0.0393 (10)	0.0020 (8)	−0.0022 (8)	−0.0062 (9)
C3	0.0287 (10)	0.0376 (10)	0.0404 (11)	0.0008 (7)	0.0016 (8)	−0.0067 (8)
C4	0.0256 (10)	0.0376 (10)	0.0428 (11)	0.0035 (7)	0.0003 (8)	−0.0074 (8)
C5	0.0280 (10)	0.0340 (9)	0.0390 (10)	0.0004 (7)	0.0000 (7)	−0.0077 (8)
C6	0.0313 (10)	0.0375 (10)	0.0383 (10)	−0.0012 (8)	0.0023 (8)	−0.0083 (8)
C7	0.0334 (11)	0.0427 (11)	0.0401 (11)	0.0018 (8)	0.0006 (8)	−0.0084 (8)
C8	0.0368 (11)	0.0525 (12)	0.0356 (11)	0.0035 (9)	−0.0004 (8)	−0.0042 (9)
C9	0.097 (2)	0.142 (3)	0.0424 (15)	−0.011 (2)	−0.0023 (14)	−0.0094 (17)
C10	0.0544 (18)	0.110 (2)	0.093 (2)	0.0092 (15)	0.0136 (15)	−0.0082 (19)
C11	0.0547 (15)	0.0755 (16)	0.0417 (12)	0.0016 (12)	−0.0043 (10)	−0.0081 (11)
N1	0.0257 (9)	0.0437 (9)	0.0473 (9)	0.0046 (7)	0.0009 (7)	−0.0097 (8)
N2	0.0496 (12)	0.0716 (13)	0.0446 (10)	−0.0024 (9)	0.0042 (8)	−0.0110 (9)
O1	0.0468 (9)	0.0897 (12)	0.0349 (8)	0.0200 (8)	−0.0010 (7)	−0.0018 (8)
O2	0.0395 (9)	0.0859 (11)	0.0401 (8)	0.0211 (8)	0.0030 (6)	−0.0069 (8)
O3	0.0372 (9)	0.0786 (11)	0.0427 (8)	0.0188 (7)	−0.0053 (6)	−0.0032 (8)
O4	0.0494 (10)	0.1019 (13)	0.0383 (9)	0.0144 (8)	0.0017 (7)	−0.0113 (9)
O5	0.0458 (10)	0.1218 (16)	0.0445 (10)	0.0002 (9)	0.0043 (7)	−0.0103 (10)

### Geometric parameters (Å, °)

**Table:**

C1—C6	1.394 (3)	C8—O1	1.304 (2)
C1—C2	1.395 (3)	C9—N2	1.447 (3)
C1—N1	1.434 (2)	C9—H9A	0.9600
C2—C3	1.389 (2)	C9—H9B	0.9600
C2—H2	0.9300	C9—H9C	0.9600
C3—C4	1.392 (3)	C10—N2	1.447 (3)
C3—C8	1.494 (3)	C10—H10A	0.9600
C4—C5	1.395 (3)	C10—H10B	0.9600
C4—H4	0.9300	C10—H10C	0.9600
C5—C6	1.387 (2)	C11—O5	1.235 (3)
C5—C7	1.492 (3)	C11—N2	1.309 (3)
C6—H6	0.9300	C11—H11	0.9300
C7—O4	1.197 (2)	N1—N1^i^	1.251 (3)
C7—O3	1.325 (2)	O1—H1	0.8200
C8—O2	1.222 (2)	O3—H3	0.8200
			
C6—C1—C2	120.34 (17)	O1—C8—C3	114.04 (17)
C6—C1—N1	124.35 (17)	N2—C9—H9A	109.5
C2—C1—N1	115.31 (17)	N2—C9—H9B	109.5
C3—C2—C1	120.22 (18)	H9A—C9—H9B	109.5
C3—C2—H2	119.9	N2—C9—H9C	109.5
C1—C2—H2	119.9	H9A—C9—H9C	109.5
C2—C3—C4	119.34 (17)	H9B—C9—H9C	109.5
C2—C3—C8	121.08 (18)	N2—C10—H10A	109.5
C4—C3—C8	119.57 (17)	N2—C10—H10B	109.5
C3—C4—C5	120.42 (17)	H10A—C10—H10B	109.5
C3—C4—H4	119.8	N2—C10—H10C	109.5
C5—C4—H4	119.8	H10A—C10—H10C	109.5
C6—C5—C4	120.25 (17)	H10B—C10—H10C	109.5
C6—C5—C7	118.99 (17)	O5—C11—N2	124.4 (2)
C4—C5—C7	120.75 (16)	O5—C11—H11	117.8
C5—C6—C1	119.40 (18)	N2—C11—H11	117.8
C5—C6—H6	120.3	N1^i^—N1—C1	113.5 (2)
C1—C6—H6	120.3	C11—N2—C10	122.1 (2)
O4—C7—O3	123.70 (19)	C11—N2—C9	121.0 (2)
O4—C7—C5	124.31 (18)	C10—N2—C9	116.8 (2)
O3—C7—C5	111.99 (17)	C8—O1—H1	109.5
O2—C8—O1	122.62 (18)	C7—O3—H3	109.5
O2—C8—C3	123.32 (18)		

Symmetry codes: (i) −*x*+3, −*y*, −*z*+1.

### Hydrogen-bond geometry (Å, °)

**Table:**

*D*—H···*A*	*D*—H	H···*A*	*D*···*A*	*D*—H···*A*
C4—H4···O3	0.93	2.38	2.704 (3)	100
C9—H9A···O5	0.96	2.37	2.763 (3)	104
O1—H1···O5^ii^	0.82	1.72	2.541 (2)	174
O3—H3···O2^iii^	0.82	1.94	2.697 (2)	154
C4—H4···O3^iii^	0.93	2.42	3.305 (2)	159
C11—H11···O2^iv^	0.93	2.58	3.240 (3)	128

Symmetry codes: (ii) *x*, *y*, *z*+1; (iii) −*x*+1, −*y*+1, −*z*+1; (iv) *x*, *y*, *z*−1.
